# *Trapa bispinosa* Roxb. Pericarp Extract Exerts 5α-Reductase Inhibitory Activity in Castrated Benign Prostatic Hyperplasia Model Mice

**DOI:** 10.3390/ijms241411765

**Published:** 2023-07-21

**Authors:** Takashi Fujita, Tomoko Aoyama, Tomohiro Uemura, Shouko Takeshita, Takuto Yamasaki, Hiroko Heijou, Koji Morimoto

**Affiliations:** 1 Department of Pharmaceutical Sciences, Ritsumeikan University, Kusatsu 525-8577, Japan; 2 Hayashikane Sangyo Co., Ltd., Yamaguchi 750-8608, Japan

**Keywords:** *Trapa bispinosa* Roxb. pericarp extract, prostate, hyperplasia, androgen receptor, 5α-reductase

## Abstract

*Trapa bispinosa* Roxb. pericarp extract (TBE) has a polyphenol-rich composition and exhibits potent antioxidant and anti-glycation activities in vitro. In the present study, we investigated the inhibitory effects of TBE on 5α-reductase in vitro using LNCaP cells and in vivo using a mouse model of castrated benign prostatic hyperplasia. TBE showed concentration-dependent inhibitory effects in the 5α-reductase (5αR) activity assay. In a reporter assay using AR-Luc/LNCaP cells, TBE inhibited the activity induced by testosterone, but not that induced by dihydrotestosterone. TBE also suppressed prostate cell proliferation, prostate-specific antigens, and transmembrane protease serine 2 expression in a castrated benign prostatic hyperplasia mouse model. In addition, ellagic acid, but not gallic acid, decreased 5αR and AR-Luc activities. Together, these results suggest a potential role for TBE in benign prostatic hyperplasia through inhibition of 5αR.

## 1. Introduction

*Trapa bispinosa* Roxb. pericarp extract (TBE) contains several gallotannins and ellagic acid derivatives [1]. The phytochemical and pharmacological activities of the pericarp, which is considered a waste or byproduct, have been extensively studied [2]. TBE is an excellent source of antioxidants and has anti-glycation activities in vitro [3,4]. TBE is enriched with gallotannin derivatives, such as eugeniin; 1,6- or 2,3-Di-O-galloyl-β-D-glucose (DGG); 1,2,3,6- or 1,2,4,6-tetra-O-galloyl-D-glucose (TGG); 1,2,3,4,6-pentagalloyl glucose (PGG); and valoneic acid dilactone [1]. The beneficial effects of the constituents of TBE have been extensively explored. For instance, the potential of gallotannins and ellagic acid to alleviate oxidative stress is well documented in several in vitro experiments [5,6]. Similarly, the antiviral potency of PGG, a highly bioavailable polyphenol, has been studied in vitro using Huh-7.5 hepatoma cells and primary human hepatocytes [7]. These studies have elucidated the underlying mechanism of action of polyphenols in vitro; however, little is known about their in vivo mechanisms of action. In addition to the constituent components of TBE, the pharmacological effects of the mixture of all components have been confirmed in humans [8]. Therefore, it is necessary not only to identify the composition but also to study the bioactivity of the mixed composition of TBE to realize its pharmacological effects.

Benign prostatic hyperplasia (BPH) is an age-related condition characterized by a wide range of symptoms related to lower urinary tract function [9]. In a healthy prostate, the androgen receptors (ARs) are normally saturated with relatively low levels of androgens and AR target gene products [10]. Prostate-specific antigen (PSA) and transmembrane protease serine 2 (TMPRSS2) are well-characterized serine proteases that are synthesized by both normal and malignant epithelial cells of the prostate and seminal glands [11,12]. Under castrated conditions, the loss of androgen activity reduces semen and prostate volume. The administration of testosterone reverses the apparent loss of urine associated with PSA levels. High PSA levels in serum and enlarged prostate volume have been implicated in the progression of BPH and prostate cancer [9].

5α-reductases (5αR) are the enzymes involved in steroid metabolism, which converts dihydrotestosterone [13,14]. The effectiveness of 5α-reductases inhibitors (5αR-Is) in reducing the cell volume in BPH is evident in previous studies [15]. Gallotannins, including eugeniin, TGG, and PGG, the constituent components of TBE, have been reported to inhibit 5αR. Among these, PGG is the most potent inhibitor of 5αR (IC_50_ = 2.5 µM) [14]. Therefore, we hypothesized that TBE, as a whole, inhibits androgen-dependent prostatic hyperplasia.

To test this hypothesis, in the present study, we investigated the effects of TBE on 5αR both in vitro and in vivo. Herein, we show for the first time that TBE as a mixture is effective against BPH.

## 2. Results

### 2.1. TBE Inhibited 5αR Activity

**Figure 1:**
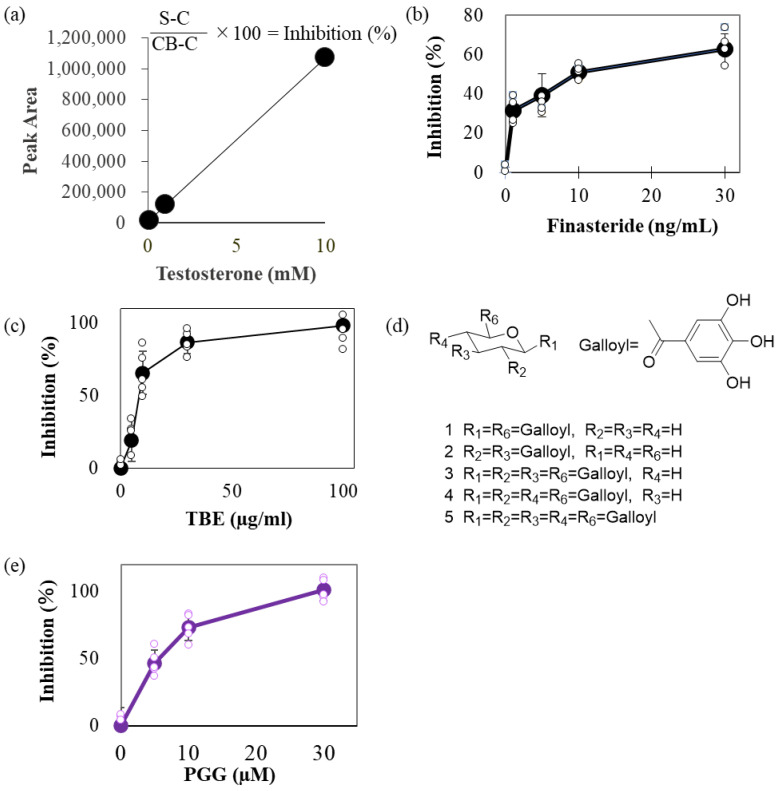
TBE inhibited 5αR activity. (**a**) Accuracy of residual testosterone measurements. The residual quantities obtained from these measurements are linear. (**b**,**c**) Inhibitory activities of finasteride (**b**) and TBE (**c**) against 5αR. Enzyme activity was estimated as described in the Section 4. (**d**) Major gallotannin constituents in TBE. Compound **5** is PGG. (**e**) Inhibitory activities of PGG against 5αR. The representative data are shown. Values are expressed as means ± SD. n = 5. TBE, *Trapa bispinosa* Roxb. pericarp extract; 5αR, 5α-reductase.

We attempted to quantify the testosterone levels using high-performance liquid chromatography (HPLC). Specific peaks were observed in a concentration-dependent manner with respect to the testosterone input (Figure 1a). To estimate 5αR-I activity modulation by TBE, we compared the effect of finasteride (Fin), a 5αR-I, and TBE on inhibiting 5αR activity. Fin and TBE inhibited 5αR activity in a concentration-dependent manner (Figure 1b,c), and the IC_50_ values of Fin and TBE were approximately 9.36 ng/mL and 20 μg/mL, respectively (Figure 1b,c). TBE contains several gallotannins, such as DGG (compounds **1** and **2**), TGG (compounds **3** and **4**), and PGG (compound **5**; Figure 1d) [1]. Among these, PGG inhibited 5αR activity at IC_50_ = 5 μM (Figure 1e).

### 2.2. TBE Inhibited AR-Luc Reporter Induced by Testosterone

**Figure 2:**
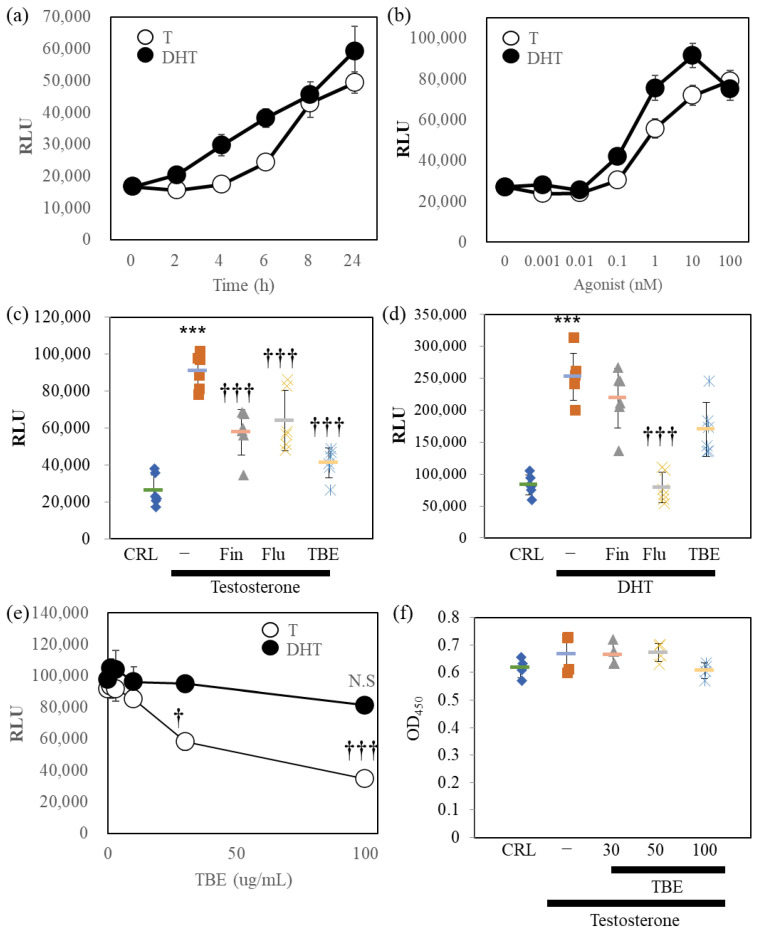
Reporter assays in AR-Luc/LNCaP cells. (**a**) AR-Luc/LNCaP cells were exposed to 10 nM testosterone (T) or 10 nM dihydrotestosterone (DHT) for the indicated durations. The cells were then harvested and lysed by 1xpassive lysis buffer. (**b**) Concentration-dependent AR-Luc reporter activity of the agonists (24 h). Luciferase activity was evaluated as described in the Section 4. n = 8. (**c**,**d**) The effect of 100 nM finasteride (Fin), 10 μM flutamide (Flu), or 100 μg/mL *Trapa bispinosa* Roxb. pericarp extract (TBE) on T- or DHT-induced AR-Luc activity. (**e**) Concentration dependence of AR-Luc activity on TBE. (**f**) Cell viability is not regulated by TBE. The representative data are shown. Values are expressed as means ± SD. n = 8. N.S., not significant. *** *p* < 0.005 compared to vehicle control (CRL), † *p* < 0.05, ††† *p* < 0.005 compared with testosterone alone. RLU, relative luciferase units.

Next, we examined the 5αR-I activity of TBE in bioassay (Figure 2). For this, we established AR-Luc–overexpressing LNCaP cells. LNCaP cells were reported as expressing cells with both 5αRs and ARs [16]. Therefore, the effects of the two AR agonists were evaluated using AR-Luc/LNCaP cells. DHT stimulated AR-Luc reporter activity, and testosterone caused delayed stimulation; both reached maximal activity at 24 h (Figure 2a). DHT and testosterone stimulated the AR-Luc cells in a concentration-dependent manner (Figure 2b). Based on these conditions, we examined the effects of pharmacological inhibitors and TBE on testosterone- or DHT-induced AR-Luc induction (Figure 2c,d). AR antagonist flutamide (Flu) inhibited testosterone or DHT-induced AR-Luc reporter activity completely, while 5αR-I Fin suppressed only testosterone-induced potentiation. TBE significantly suppressed testosterone-induced AR-Luc reporter activity without inducing cytotoxicity (Figure 2e,f).

### 2.3. TBE Ameliorated Testosterone-Induced Prostatic Gland Formation and Increased Proliferation in Castrated BPH Mice

**Figure 3:**
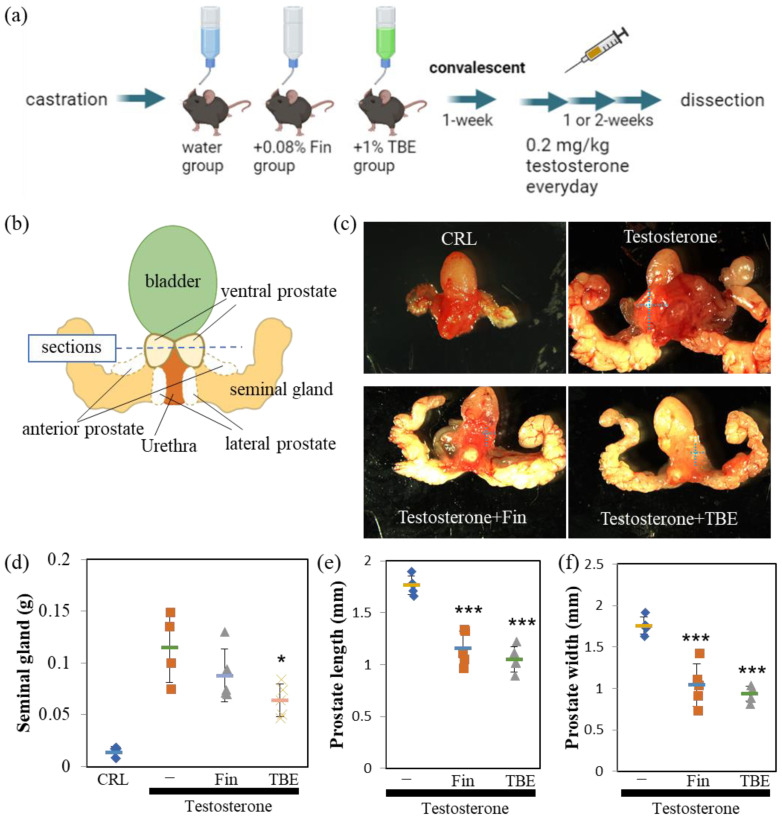
Finasteride or TBE ameliorated BPH in mice. (**a**) Schedule of the procedures. No testosterone was administered to the castration-only control group. (**b**) Image of the urinary structure. Horizontal cross-sections are obtained along the dashed line. (**c**) Finasteride (Fin) or *Trapa bispinosa* Roxb. pericarp extract (TBE) ameliorates benign prostatic hyperplasia (BPH) in mice. Dotted crosses indicate the lengths of the major and minor axes of the ventral prostate (width), as summarized in (**d**–**f**). Fin and TBE reduced testosterone-induced seminal gland weight gain (**d**) and ventral prostate size (**e**,**f**). Values are expressed as means ± SD. n = 4–5. * *p* < 0.05, *** *p* < 0.005 compared with control (testosterone control).

Since the in vitro analysis showed that TBE had 5αR inhibitory activity, we evaluated the effectiveness of TBE in a BPH mouse model (Figure 3a). The perineal regions were isolated and photographed using a stereomicroscope (Figure 3b,c). Castration resulted in involuted seminal glands, wherein seminal glands did not develop in the absence of testosterone (Figure 3c,d). By administering an appropriate concentration of testosterone to castrated mice intraperitoneally, the size of seminal and prostate glands developed up to normal mice level and was sufficient for analysis (Figure 3b,c upper). Moreover, 5αR-I Fin tended to inhibit the increase in the weight of seminal and size of ventral prostate glands (Figure 3c–f). Similarly, TBE administration inhibited the testosterone-induced increase in the weight of seminal and length and width of ventral prostate glands (Figure 3c–f).

**Figure 4:**
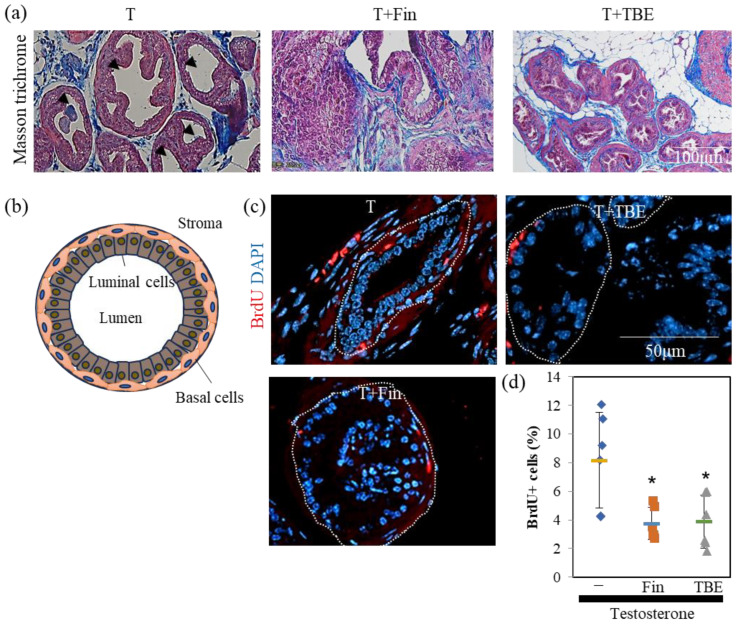
Histological analysis of benign prostrate hyperplasia mice. (**a**) Histological differences in ventral prostate gland. Paraffin sections were stained with Masson trichrome as described in Section 4. Ductal structures are highly developed in the glandular structures of testosterone control. Arrowheads indicate canalization. Representative images are shown. (**b**) Schematic diagram of glandular structure. Cells mature toward the lumen. Lumenal cells aggregate to form a lumen, which then forms a larger lumen. (**c**,**d**) Fin and *Trapa bispinosa* Roxb. pericarp extract (TBE) inhibits T-induced prostate cell proliferation. Bromodeoxyuridine (BrdU^+^) cells were counted in five sections. Representative images were shown in (**c**) and summarized in (**d**). The measurement of BrdU positivity in basal cells among glandular structures is described in Section 4. The area enclosed by the dotted line shows the glandular structure. Values are expressed as means ± SD. n = 5. * *p* < 0.05, compared to T.

**Figure 5:**
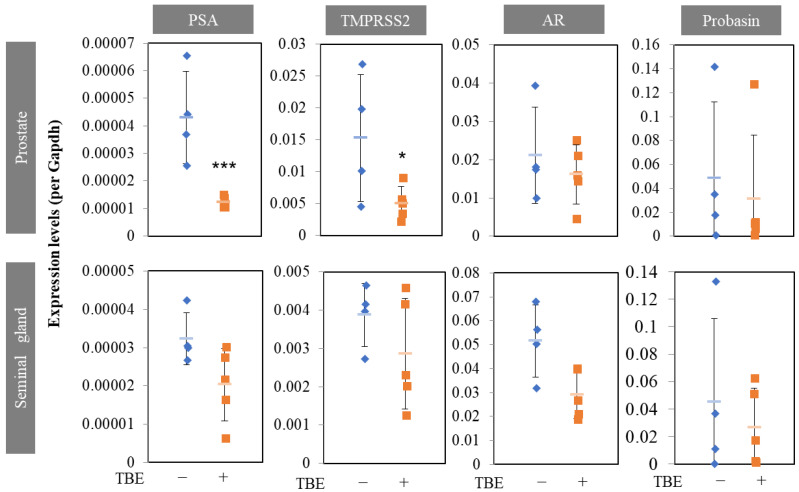
Finasteride or TBE suppressed PSA and TMPRSS2 expressions in BPH mice. *Trapa bispinosa* Roxb. pericarp extract (TBE) regulates PSA or TMPRSS2 expression in the prostate and seminal glands. The prostate and seminal glands were excised under a stereomicroscope and immediately homogenized in Sepasol. Seminal gland samples were prepared from the apical regions. Values are expressed as means ± SD. n = 4–5. * *p* < 0.05, *** *p* < 0.005 compared with testosterone. AR, androgen receptor.

Next, we examined the effect of TBE on these mechanisms using histological analysis. Sections were prepared along the lines shown in Figure 3b. Masson’s trichrome staining revealed an involuted prostate (Figure 4a). Morphologically involuted prostate cells were observed, which correlated with the TBE-induced reduction in ventral prostate size. Testosterone forms a lumen in the secretory gland structure (canalization). Canalization occurs before epithelial cell differentiation and thus indicates a mature gland [17,18]. Canalization and lumen formation abundantly occurred in testosterone (T)-administered mice, whereas Fin and TBE suppressed them. Sections were examined for prostate cell proliferation using the fluorescence immunohistochemistry technique (Figure 4c). Basal cells proliferate in the direction of lumen formation. Thus, we measured the proliferation rate of basal cells. Since the morphology of the nuclei of basal cells differs from that of lumenal cells, we measured only the flat nuclei. The percentage of BrdU⁺ cells in the basal cells of the secretory glands was measured, and treatment with Fin or TBE inhibited cell proliferation (Figure 4c,d). Treatment with Fin or TBE suppressed PSA (also called Klkb1 in mouse), and TMPRSS2 expression qPCR analysis demonstrated that prostate PSA and TMPRSS2 expression were downregulated by TBE (Figure 5). Similarly, AR expression levels and AR-target probasins were not regulated by TBE (Figure 5).

### 2.4. Ellagic Acid Inhibited 5αR Both In Vitro and In Vivo

**Figure 6:**
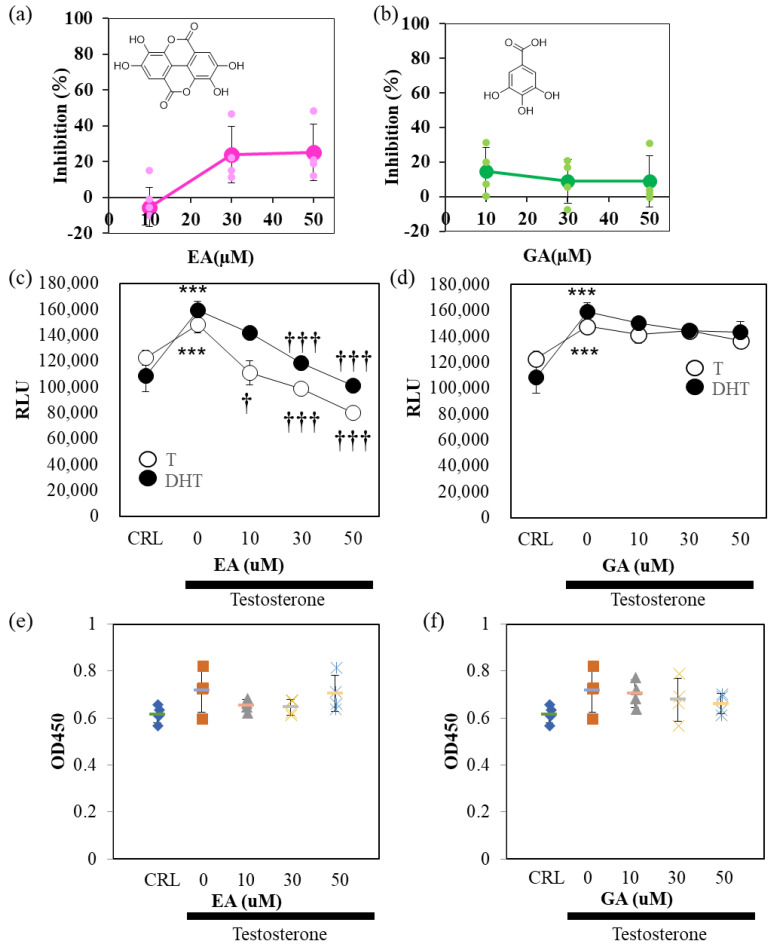
EA, but not GA, inhibits 5αR activity. Ellagic acid (EA) (**a**), but not gallic (GA) (**b**), inhibits 5α- reductase (5αR) activity. The assay conditions are shown in Figure 1. (**c**,**d**) EA, but not GA inhibits 5αR activity in AR-Luc/LNCaP cells. EA, but not GA, inhibits testosterone (T)- and dihydrotestosterone (DHT)-induced AR-Luc reporter activity in a concentration-dependent manner. (**e**,**f**) Cell viability is not affected by either EA or GA. The representative data are shown. Values are expressed as means ± SD. n = 8. *** *p* < 0.005 compared to vehicle control (CRL), † *p* < 0.05, ††† *p* < 0.005 compared with T alone.

**Figure 7:**
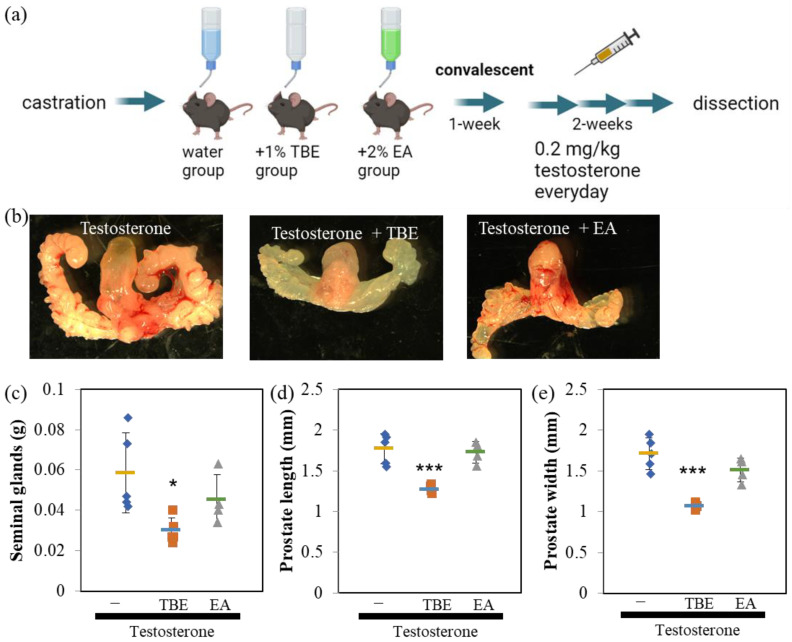
EA failed to ameliorate BPH model mice. (**a**) Schedule of the experimental procedures. (**b**–**e**) *Trapa bispinosa* Roxb. pericarp extract (TBE), but not ellagic acid (EA), ameliorates benign prostatic hyperplasia (BPH) in mice. EA does not reduce the seminal gland weight (**c**) and ventral prostate size (**d**,**e**) induced by testosterone. Values are expressed as means ± SD. n = 4–5. * *p* < 0.05, *** *p* < 0.005 compared with testosterone.

Finally, we examined the effect of TBE metabolites, EA, and gallic acid (GA), on 5αR activity (Figure 6). In the cell-free system, EA, but not GA, weakly inhibited 5αR activity (Figure 6a,b). The suppression rate was approximately 20%. In AR-Luc/LNCaP cells, EA, but not GA, inhibited 5αR activity in a concentration-dependent manner (Figure 6c,d). The AR-Luc inhibitory activity of EA was partial, and the induction of AR-Luc by DHT was suppressed to control levels without cellular toxicity (Figure 6e,f). To confirm these in vitro results, the cells were tested in a mouse model of BPH (Figure 7). EA was administered at a 2% concentration by auto-withdrawal for 1 week during the postoperative convalescent period (Figure 7a). EA was administered at a concentration of 2% by auto-withdrawal during the postoperative convalescent period. The results of this study showed that TBE reproducibly decreased seminal gland weight and ventral prostate size, whereas EA had negligible effects (Figure 7b,c).

## 3. Discussion

Herein, we examined the antioxidative and antiglycative properties of TBE, a natural product. TBE has been recognized as a medicinal and nutritional food in Asia. Several studies have explored the pharmacological potency of its constituent bioactive materials against several complications. Several natural products have been reported to exhibit 5αR inhibitory activity; however, almost no compounds demonstrating in vivo efficacy have been identified [19,20]. We showed evidence that TBE improved BPH by inhibiting 5αR both in vitro and in vivo.

TBE showed 5αR inhibitory activity in vitro (Figure 1 and Figure 2). In most cases, androgenic responses were weak or undetectable in cultured cells despite AR expression, at least in LNCaP cells. In three-dimensional cultures, sex hormones, including testosterone, may mimic in vivo responses [21]. Studies have shown that GA does not exert 5αR inhibitory effects but is effective against prostate cancer [22]. TBE showed anti-AR activity in the reporter assay (Figure 2). The effect of DHT, a direct androgen ligand, was also significantly suppressed by Flu. TBE tended to inhibit the effects of DHT, which may be due to the inhibitory effects of EA in TBE on non-specific reactions (Figure 2d and Figure 6c). In other words, it is conceivable that TBE may effectively improve BPH through multiple mechanisms, including antioxidative effects. Therefore, further studies are warranted.

Androgens play an important role in human BPH. Despite this, 5αR-I is particularly effective in reducing prostate volume [9,15]. For this reason, the castrated BPH mouse model has been widely used in studies measuring the effects of 5αR-I. In the in vivo study, the effects of TBE were examined for the effects of Fin, which suppressed or tended to suppress the expression of two target genes of ARs, PSA, and TMPRSS2 (Figure 3, Figure 4 and Figure 5). Therefore, organogenesis occurs based on weight and size. Although several natural products have been reported to exhibit 5αR inhibitory activity, almost no compounds have been identified that have demonstrated in vivo efficacy [19,20]. We showed evidence that TBE improved BPH by inhibiting 5αR both in vitro and in vivo. Probasin is a marker of prostate differentiation and is a target gene for ARs. Probasin expression is downregulated in prostate epithelial-specific AR knockout mice [23]. An AR-independent transcriptional regulatory region exists in the probasin promoter [24]. Taken together, PSA is more AR-dependent than probasin, and TBE repressed PSA more strongly than probasin.

The 5αR-I activities of the metabolites of TBE, including GA and EA, were investigated (Figure 6). EA, but not GA, partially inhibited 5αR without inducing cell toxicity (Figure 6). Because EA inhibited not only testosterone but also DHT-induced AR-Luc activity, it is unlikely that the effect of EA is due to selective inhibition of 5αR, as described above. EA may have multiple points of action. PGG effectively inhibits 5αR in vitro but is not taken up into circulation in an intact form because of its low bioavailability [5,6]. When DGG, TGG, and PGG enter the body, they are hydrolyzed, and GA is released and absorbed. GA is a bioactive compound with antioxidant, anticancer, anti-inflammatory, and antimicrobial properties [25]. However, 5αR-I activity inhibition was observed with EA, but not with GA, in vitro (Figure 6). Hence, it is possible that other components of TBE, including EA, besides GA, contribute to the increase in the absorption of the active ingredient to improve BPH. TBE contains gallotannins that exert 5αR inhibitory activity [1,5,6]. However, since abundant constituent EA alone did not show more BPH inhibition than TBE (Figure 7), other compositions of TBE may have modulated the bioavailability of 5αR inhibitory constituents. Further investigations regarding this aspect are needed. Nevertheless, the present study provides evidence for the potential of TBE, a mixture comprising constituent components, including EA, GA, and gallotannins, to inhibit 5αR activity.

Based on both in vitro and in vivo evaluations, we showed that TBE prevents BPH by inhibiting 5αR. TBE reliably inhibits 5αR, suggesting the potency of TBE against BPH as well as in androgenic alopecia.

## 4. Materials and Methods

### 4.1. Reagents

TBE was prepared as previously described [8,26]. In brief, *Trapa bispinosa* Roxb. peels were dried and extracted using hot water. Dextrin was added to the extract in a ratio of 67:33 of dry weight, and the solution was spray-dried to obtain TBE. The sample solutions for subsequent measurements were prepared by dissolving TBE in distilled water. Eugeniin was purchased from Kishida Chemicals (Tokyo, Japan). PGG was purchased from MedChem Express (Monmouth Junction, NJ, USA) or synthesized from glucose with 3,4,5-tris (benzyloxy) benzoic acid by condensation using 1-ethyl-3-(3-dimethylaminopropyl) carbodiimide hydrochloride under a nitrogen atmosphere in dichloromethane. Unless otherwise stated, reagents were purchased from Nacalai Tesque (Kyoto, Japan) or FUJI Film Wako Pure Chemical Industries (Osaka, Japan).

### 4.2. High-Performance Liquid Chromatography (HPLC) Analysis

HPLC analysis to estimate 5αR-I activity was performed as described by Morikawa et al. with several modifications [27]. Briefly, the S9 rat liver homogenate (Oriental Yeast Co., Ltd., Tokyo, Japan) as an enzyme solution, with or without a test sample, was pre-incubated in potassium phosphate buffer (40 mM, pH 6.5) containing nicotinamide adenine dinucleotide phosphate (NADPH) (10 nmol, Oriental Yeast Co., Ltd., Tokyo, Japan) at 25 °C for 30 min. After the addition of 0.2 mg/mL testosterone to the mixture and allowed to react in a 37 °C bath for 30 min. Afterward, the reaction solution was transferred to a tube and extracted with ethyl acetate (EtOAc). The mixture was centrifuged (760× *g*, 10 min), and aliquots of each EtOAc phase were transferred to glass microtubes. The solvent in the tubes was evaporated and the residue was dissolved in 300 μL of acetonitrile (Kanto Chemical Co., Ltd., Tokyo, Japan). The prepared sample (200 μL) was injected into the HPLC system (LC-20AB Prominence HPLC system; Shimadzu Co., Kyoto, Japan) fitted with SIL-20AC sampler, SPD-M20A PDA detector (242 nm), andUnison UK-C18 column (Imtakt Corp., Kyoto, Japan, 33 μm particle size, 4.6 mm i.d. × 150 mm length). The separation was carried out under the following condition: column temperature, 40 °C; mobile phase, acetonitrile-H_2_O (50:50, *v*/*v*); flow rate, 0.4 mL/min; retention time, 15 min for testosterone. The accuracy of the data was determined based on the linearity of testosterone peaks (Figure 1a). The inhibition rate against 5αR was calculated using the following formula:

$$\begin{array}{l} 5\alpha R\;inhibition\;rate=\\ \frac{Sample\;testosterone\;residual\;amount\;(peak\;area;\;S)-Control\;testosterone\;residual\;amount\;(peak\;area;\;C)}{Control\;blank\;testosterone\;residual\;amount\;(peak\;area;\;CB)-C} \end{array}$$

### 4.3. Reporter Cells and Cell Viability Test

LNCaP.FGC (LNCaP) cells (RCB2144) were purchased from Riken Cell Bank (Ibaraki, Japan). pGL4.36 [luc2P/MMTV/Hygro] vector was purchased from Promega (Madison, WI, USA). The murine mammary tumor virus long-terminal repeat sequence function was used as androgen and glucocorticoid sensors [28]. Stably overexpressing LNCaP cells were generated by transfecting the reporter vector with polyethyleneimine. Clones were selected using 100 µg/mL hygromycin and isolated independently, and three clones were obtained. Reporter activity was evaluated using the One-Glo Luciferase Assay System (Promega). The luminescence activity was measured using a GloMax^®^ Discover Microplate Reader (Promega). However, the following points should be noted with caution: within 24 h of seeding, cells in the exponential growth phase showed almost no response to testosterone or DHT; therefore, treatment was initiated after incubation of ≥48 h or more.

For cell viability testing, Cell Counting Reagent SF (Nacalai Tesque) was used according to the manufacturer’s protocol as previously described [29]. Briefly, confluent cells in 96-well plates were treated with reagents for 24 h, then 10 μL of WST-8 reagent was added to each well. After 90 min, cell viability was determined by measuring the absorbance at 450 nm.

### 4.4. Animal Experiments

Seven-week-old male mice were obtained from a commercial supplier (SLC Japan, Shizuoka, Japan) and housed for 1 week in a semi-barrier animal room (12:12 light/dark cycle, ad libitum feeding). The mice were divided into three groups: control, 2.0 mg/kg testosterone, TBE plus testosterone (testosterone + TBE), and finasteride (Fin) plus testosterone (testosterone + Fin) or ellagic acid (EA) plus testosterone (testosterone + EA). Castration was performed as described by Lofgren et al. [30]. Briefly, anesthetized mice were disinfected with povidone-iodine and ethanol, and a sterile scissor incision was made in the lower abdomen to expose the testes. After double ligation of the testicular artery and vein with a melting suture (Akiyama Medical Co., Ltd., Tokyo, Japan), the testicles were returned to the abdomen, and the open abdomen was sutured. Then, 0.2 mL of 5 mg/mL penicillin G per day was injected intraperitoneally for 3 days after surgery, and 0.08% Fin, 1% TBE, or 2% ellagic acid by free drinking water in 1% carboxymethylcellulose (CMC) was provided until dissection. After castration, the mice were allowed to recover for 1 week. Approximately 0.2 mL of 0.02 mg/mL testosterone in 1% CMC was injected intraperitoneally daily into all three groups. Twenty-four hours before dissection, 100 mg/kg bromodeoxyuridine (BrdU) was injected intraperitoneally. The isolated organs were observed using a Leica MZ10F/DFC7000T stereomicroscope (Leica Microsystems, Mannheim, Germany), and the size of the ventral prostate was measured from the acquired images using LAS ver.4.12 application (Leica Microsystems). Seminal gland weights were measured after removing the anterior, lateral, and dorsal prostate under a stereomicroscope.

### 4.5. Histology and Immunohistochemical Analysis

The prostates, with other urinary systems, were fixed in 10% neutral buffered formalin and embedded in paraffin blocks. Masson’s trichrome staining was performed as previously described [31]. After deparaffinization, the slides were washed with 10% potassium dichromate/10% trichloroacetic acid, and the nuclei were stained with iron hematoxylin. After staining with 2.5% phosphotungstic acid/2.5% phosphomolybdic acid and 0.75% Orange G solution, the cells were washed with 1% acetic acid. The cytoplasm was stained with ponsoxylidine/acid fuchsin/azophloxine solution and washed with 1% acetic acid. The specimen was then stained with 2.5% phosphotungstic acid, washed with 1% acetic acid, and the connective tissue was stained with aniline blue. The stained specimens were then washed, dehydrated, and mounted using a soft mount (FUJI Film Wako Pure Chemical Industries).

Immunohistochemical analysis was performed as previously described [29]. Briefly, the samples were incubated with anti-BrdU (66241) (Proteintech, Rosemont, IL, USA) as primary antibodies, and Alexa Fluor 555-conjugated anti-mouse immunoglobulin G (Thermo Fisher Scientific, Waltham, MA, USA) as secondary antibodies. The slides were mounted in Fluor-KEEPER anti-fade reagent with 4′,6-diamidino-2-phenylindole (DAPI; Nacalai Tesque). Observations and scoring were performed using a BX51/DP74 fluorescence microscope (Olympus, Tokyo, Japan) and the CellSens software V4.2 (Olympus). To monitor prostate cell proliferation, the rate of BrdU incorporation was determined by scoring BrdU-positive cells per total prostate basal cells (BrdU^+^ plus DAPI^+^). Three sections per mouse were stained, and images were taken at 200× magnification. The proliferation rates were calculated from the results of at least five fields of view.

### 4.6. RNA Isolation and Quantitative Real-Time Polymerase Chain Reaction (QPCR)

Total RNA was isolated using Sepasol^®^ (Nacalai Tesque, Kyoto, Japan) and isolated total RNA was used to synthesize complementary DNA using RevaTra Ace^®^ (TOYOBO, Shiga, Japan), as previously described [27]. Quantitative real-time PCR analysis based on the intercalation of SYBR Green (Nippon Genetics Co., Ltd., Tokyo, Japan) was performed as previously described [18]. The following primer sequences were used: Gapdh-F 5′-ATGTGTCCGTCGTGGATCTGA-3′, Gapdh-R 5′-CCTTCTCCATGGTGGT-3′ (NM_001289726, 145 bp), Klkb1/PSA-F 5′-TGGTCGCCAATGGGTACTG-3′, Klkb1/PSA-R 5′-ATATACGCCACACATCTGGATAGG-3′ (NM_008455, 71 bp), TMPRSS2-F 5′-AAGTCCTCAGGAGCACTGTGCA-3′, TMPRSS2-R 5′-CAGAACCTCCAAAGCAAGACAGC-3′ (NM_015775, 116 bp), AR-F 5′-TTGCAAGAGAGCTGCATCAGTT-3′, AR-R 5′ACTGTGTGTGGAAATAGATGGGC-3′ (NM_013476, 153 bp), Probasin-F 5′-GGTCATCATCCTCCTGCTCA-3′, Probasin-R 5′-AGGCCCGTCAATCTTCTTTTT-3′ (NM_017471, 79 bp). The housekeeping gene Gapdh served as an internal control and was used to normalize the differences in the input RNA.

### 4.7. Statistical Analysis

Data are expressed as mean ± standard deviation (SD). Significance was tested using a one-way analysis of variance (ANOVA) followed by post hoc Bonferroni test or Student’s *t*-test. A *p*-value of less than 0.05 was considered statistically significant.

## Data Availability

Not applicable.
